# The German version of the Expanded Prostate Cancer Index Composite (EPIC): translation, validation and minimal important difference estimation

**DOI:** 10.1186/s12955-018-0859-1

**Published:** 2018-02-20

**Authors:** Martin H. Umbehr, Lucas M. Bachmann, Cedric Poyet, Peter Hammerer, Johann Steurer, Milo A. Puhan, Anja Frei

**Affiliations:** 1 Department of Urology, City Hospital Triemli of Zurich, Birmensdorferstrasse 497, 8063 Zurich, Switzerland; 2 Horten Centre of patient orientated research and knowledge transfer, University of Zurich, Zurich, Switzerland; 3 Medignition AG, Zurich, Switzerland; 4 Department of Urology, University Hospital of Zurich, Zurich, Switzerland; 5 Clinic of Urology, Städtisches Klinikum Braunschweig, Braunschweig, Germany; 6 Epidemiology, Biostatistics and Prevention Institute, University of Zurich, Zurich, Switzerland

**Keywords:** EPIC, Prostate Cancer, Quality of Life Assessment

## Abstract

**Background:**

No official German translation exists for the 50-item Expanded Prostate Cancer Index Composite (EPIC), and no minimal important difference (MID) has been established yet. The aim of the study was to translate and validate a German version of the EPIC with cultural adaptation to the different German speaking countries and to establish the MID.

**Methods:**

We translated and culturally adapted the EPIC into German. For validation, we included a consecutive subsample of 92 patients with localized prostate cancer undergoing radical prostatectomy who participated the Prostate Cancer Outcomes Cohort. Baseline and follow-up assessments took place before and six weeks after prostatectomy in 2010 and 2011.

We assessed the EPIC, EORTC QLQ-PR25, Feeling Thermometer, SF-36 and a global rating of health state change variable. We calculated the internal consistency, test-retest reliability, construct validity, responsiveness and MID.

**Results:**

For most EPIC domains and subscales, our a priori defined criteria for reliability were fulfilled (construct reliability: Cronbach’s alpha 0.7–0.9; test-retest reliability: intraclass-correlation coefficient ≥ 0.7). Cross-sectional and longitudinal correlations between EPIC and EORTC QLQ-PR25 domains ranged from 0.14–0.79, and 0.06–0.5 and 0.08–0.72 for Feeling Thermometer and SF-36, respectively. We established MID values of 10, 4, 12, and 6 for the urinary, bowel, sexual and hormonal domain.

**Conclusion:**

The German version of the EPIC is reliable, responsive and valid to measure HRQL in prostate cancer patients and is now available in German language. With the suggested MID we provide interpretation to what extent changes in HRQL are clinically relevant for patients. Hence, study results are of interest beyond German speaking countries.

**Electronic supplementary material:**

The online version of this article (10.1186/s12955-018-0859-1) contains supplementary material, which is available to authorized users.

## Background

Prostate cancer is one of the most prevalent cancers in men. Due to the growing number of long-term survival rates [1], maintenance of health-related quality of life (HRQL) is crucial for prostate cancer patients and should be taken carefully into account when planning individual treatment strategies [2]. Data on effects and side effects [3] of different treatment modalities on HRQL are of great value to guide the physicians’ advice and patients’ decision on choice of treatment. Therefore, patient-reported outcome measures are required that appropriately assess relevant aspects of HRQL and that have well-established psychometric properties, i.e. the measures should be reliable, valid and able to detect patient important changes in HRQL over time (minimal important difference, MID). A well-established MID of an instrument is particularly important for clinicians and researchers because it shows whether a specific change score derived from assessments before and after an intervention actually reflects a difference that is relevant for the patient.

Several generic and disease specific HRQL instruments have been introduced and recommended for prostate cancer patients so far [4]. One of the most established and frequently used instrument focussing on disease-specific aspects of prostate cancer and its therapies is the 50-item Expanded Prostate Cancer Index Composite (EPIC) [5]. The EPIC was evolved from the UCLA-Prostate Cancer Index (UCLA-PCI) [6] by an expert panel. Originally developed in the U.S. in English language, the EPIC has been translated and validated into several other languages such as Korean [7], Japanese [8], Brazilian [9] and Spanish [10]. In addition, two short form versions have been introduced, a 26-item (EPIC-26) [11] and a 16-item (EPIC-CP) [12] version. Although short versions of questionnaires are generally useful in clinical practice, a loss of precision in the assessment is inevitable when using them compared to the extensive versions. Therefore, the original and extensive EPIC 50-item version is valuable whenever detailed assessment is required. Compared to the other frequently used instrument, the European Organization for the Research and Treatment of Cancer Quality of Life Questionnaire–Prostate 25 (EORTC QLQ-PR25) tool [13], the EPIC seems to provide a better balanced performance to assess more in depths the various side effects independent of treatment modality finally chosen.

To our knowledge, no MID of the original EPIC 50-item version has been established so far, and, except for a translation of the EPIC-26 [14], no validated German version exists. Since over 100 million people worldwide are native German speakers, a German version of this important instrument is of great value. The aims of this study were to translate and validate the German version of the EPIC with cultural adaptation to the different German speaking countries Germany, Austria and Switzerland, and to establish the MID for each domain of the instrument.

## Methods

The unabridged version of the Material and methods section is presented in Additional file 2.

### The Expanded Prostate Cancer Index Composite (EPIC)

The EPIC consists of 50 items with Likert type response options contributing to the four domains *Urinary*, *Bowel*, *Sexual* and *Hormonal*. Each domain is divided into the two subscales *Functional* and *Bother* assessing symptoms severity and the extent of symptom-related HRQL impairment. The urinary domain is additional divided into the two distinct *Incontinence* and *Irritation*/*Obstruction* subscales. Domains and subscales are presented in 0–100 scales with higher scores representing better HRQL.

### Translation and cultural adaptation

**Fig. 1:**
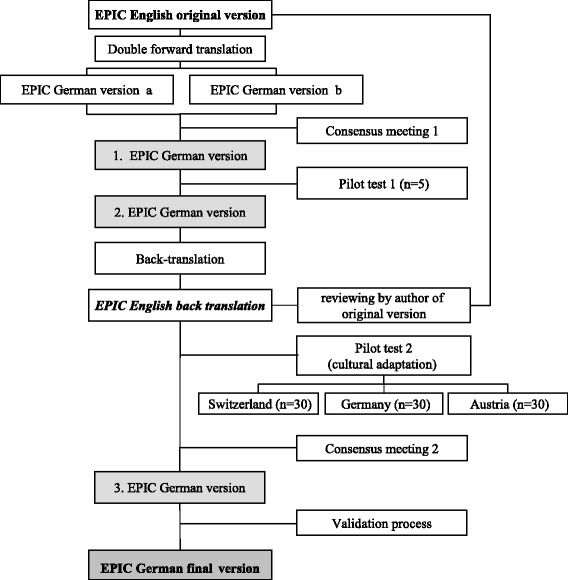
Translation process. The progress of for- and backward translation into German language including two rounds of pilot testing as well as cultural adaption

The translation and cultural adaptation process was based on the recommendations of the ISPOR Task Force international expert group [15] and followed a sequential forward and backward translation approach (Fig. 1). Two professional translators translated the original English EPIC version into German. In a consensus meeting, five experts assessed the consistency of the translations, judged their face validity and agreed on a first German version. This first version was pretested in cognitive debriefings in five prostate cancer patients who agreed on a second German version. A professional translator translated this version back into English language which was then presented to the author of the original English version [5]. The second German version then was pretested in the three German speaking countries (Austria, Germany and Switzerland), each of them within thirty patients to assess the need for cultural adaptation. Finally, the experts agreed on a third, final version of the German version of the EPIC (Additional file 2).

### Study population and study design

For the validation study, we recruited a subsample of patients who participated the Prostate Cancer Outcomes Cohort (proCOC) [16] who had a diagnosis of localized prostate cancer and underwent robotic radical prostatectomy within the Department of Urology of the University Hospital of Zurich between November 2008 and December 2010. The local Ethical Committee of the Canton of Zurich approved the study.

Baseline assessments took place after the diagnosis and before radical prostatectomy and included assessments of the EPIC and the validation instruments (internal consistency and cross-sectional construct validity). To assess test-retest reliability, a subgroup of participants completed the EPIC a second time one to two weeks later, before initiation of treatment. Follow-up assessments were conducted six weeks after treatment (longitudinal construct validity, responsiveness and MID).

### Validation instruments

The European Organization for the Research and Treatment of Cancer Quality of Life Questionnaire–Prostate 25 (EORTC QLQ-PR25) [13] is a prostate specific additional tool to the general quality of life instrument EORTC QLQ-C30 [17]. It includes 25 items with a 4-point Likert scale that contribute to the sexual activity and sexual functioning (scores 0–100; higher scores = higher level of functioning) and urinary symptoms, bowel symptoms and hormonal treatment-related symptoms scales (scores 0–100, higher score = higher level of problems) [13]. For the EORTC-QLQ-C30, a MID of 5–10 has been established [17, 18], no MID has been specifically reported so far for the prostate additional tool.

The Feeling Thermometer assesses generic health status on an analogue scale presented as a thermometer with 100 marked intervals (0 = dead to 100 = perfect health), a MID of 5 has been reported [19–21].

The SF-36 version 2.0 is a generic quality of life instrument that consists of 36 items describing 8 domains (scores 0–100; higher scores = better perceived state of health) [22, 23]. MID between 7 and 12 have been suggested specifically for prostate cancer survivors [24].

The patients also rated the global change of their health state since baseline (after treatment) on a 5-point Likert scale at follow up, ranging from − 2 (my health state worsened much) to + 2 (my health state improved much).

### Statistical analysis

We assessed internal consistency of the EPIC scores by Cronbach’s alpha (adequate internal consistency a priori defined: 0.7–0.9) and test-retest reliability by intraclass correlation coefficients (ICC; a priori defined: ≥0.70 [25]). To assess cross-sectional and longitudinal construct validity we used Pearson or Spearman’s rank correlation coefficients at baseline and at 6 weeks follow-up between the EPIC domain scores and the validation measures or the change sores, respecitvely. We a priori expected strong correlations (≥0.5) between the EPIC and the corresponding EORTC QLQ-PR25 scores and moderate correlations (0.3–0.5) between the EPIC and the Feeling Thermometer and selected SF-36 domain scores.

To quantify responsiveness, we assessed the standardised response mean (SRM) as the mean change score divided by SD of change score (a priori stronger effect sizes in urinary/sexual compared to bowel/hormonal domains expected). We established the MID by triangulation and used both anchor-based approaches (using EPIC domain change scores against the anchors “worsened” and “remained the same” of the global rating of health state chance variable) and distribution-based approaches (standard error of measurement [SEM], Cohen’s effect size, empirical rule effect size) [26]. Analysis were performed using STATA version 13 [27].

## Results

Ninety two consecutive participants of the proCOC study with localized prostate cancer and a mean age of 62.3 ± 7.1 years participated in the validation study and completed baseline assessments (before treatment) and 6 week follow-up assessments (after treatment). Mean scores of the EPIC summary domains and subscales and the validation instruments at baseline and at 6 weeks follow-up after robotic assisted radical prostatectomy and changes from baseline to follow-up are presented in Table 1. A subsample of 44 participants of proCOC with a mean age of 62.5 ± 7.4 years completed the EPIC a second time before treatment to assess test-retest reliability, on average with 10.9 ± 13.3 days between the assessments.

**Table 1:** Mean scores of the EPIC summary domains and subscales and the validation instruments at baseline and at 6 weeks follow-up after robotic assisted radical prostatectomy and changes from baseline to follow-up

Measurement	Baseline (*n* = 92)^a^	6-weeks follow-up (n = 92)^a^	Change from baseline to follow-up (n = 92)^a^
EPIC domain-specific summary scores			
Urinary	90.0 (11.9)	61.7 (18.5)	−28.3 (18.7)
Bowel	94.4 (7.8)	87.8 (11.6)	−6.8 (11.0)
Sexual	61.1 (20.4)	17.5 (12.1)	−44.0 (23.0)
Hormonal	90.5 (11.3)	87.2 (13.2)	−3.3 (12.9)
Urinary subscales			
Function	96.2 (9.6)	54.6 (21.8)	−41.6 (22.1)
Bother	85.6 (15.2)	66.8 (18.3)	−18.8 (19.5)
Incontinence	94.8 (13.4)	37.2 (27.4)	−57.6 (27.4)
Irritation/obstruction	88.8 (12.9)	77.0 (17.4)	−11.2 (18.8)
Bowel subscales			
Function	93.8 (8.6)	88.3 (10.7)	−5.7 (11.4)
Bother	95.1 (8.9)	87.4 (14.4)	−7.7 (13.3)
Sexual subscales			
Function	56.7 (21.9)	9.1 (11.3)	−47.1 (22.4)
Bother	72.5 (23.8)	35.9 (28.2)	− 37.2 (32.8)
Hormonal subscales			
Function	90.1 (12.7)	85.0 (14.9)	−5.1 (15.4)
Bother	90.8 (11.3)	88.8 (12.8)	−1.9 (12.3)
EORTC QLQ-PR25 symptom scales			
Urinary symptoms	17.0 (16.0)	40.1 (20.8)	22.9 (19.4
Bowel symptoms	4.5 (7.9)	7.8 (9.5)	2.6 (10.3)
Hormonal treatment-related symptoms	6.5 (9.7)	11.9 (12.0)	4.7 (11.8)
EORTC QLQ-PR25 functional scales			
Sexual activity	59.0 (25.4)	22.5 (24.6)	−25.0 (30.6)
Sexual functioning	79.5 (19.0)	38.8 (19.4)	− 40.4 (25.6)
Feeling Thermometer	79.8 (15.5)	71.01 (17.3)	−6.4 (19.7)
SF-36			
Physical Functioning	96.7 (6.5)	77.3 (19.8)	−19.4 (20.1)
Role-Physical	89.3 (19.9)	58.3 (27.0)	− 31.0 (29.8)
Bodily Pain	88.9 (17.9)	74.8 (27.4)	−17.2 (31.1)
General Health	74.7 (16.5)	71.1 (17.0)	−3.5 (16.1)
Vitality	38.6 (8.9)	31.3 (12.3)	−7.2 (11.5)
Social Functioning	85.3 (20.7)	72.3 (28.2)	−13.0 (31.1)
Role-Emotional	88.9 (18.9)	74.8 (27.4)	−14.1 (26.9)
Mental Health	30.9 (6.7)	29.9 (8.3)	−1.0 (7.5)

*Abbreviations: EPIC* Expanded Prostate Cancer Index Composite, *ERTC QLQ-PR25* European Organization for the Research and Treatment of Cancer Quality of Life Questionnaire–Prostate 25 t

^a^Missing values: All EPIC scores: 0–2, except for Sexual function subscale: Baseline = 5/follow-up = 4/change = 8; EORTC QLQ-PR25: Urinary symptoms: 5/3/8; Bowel symptoms: 8/3/11; Hormonal treatment related symptoms: 8/2/10, Sexual activity: 7/3/10, Sexual functioning: 23/57/62; Feeling Thermometer: 24/9/26; SF-36: no missing values

### Internal consistency and test-retest reliability

Characteristics of the EPIC domain and subscale scores and the results on internal consistency and reproducibility are presented in Table 2. In general, urine and sexual domain scores of the patients decreased to a greater extent from baseline to 6 weeks follow-up than did bowel and hormonal domain scores. For the majority of the domain and subscale scores, Cronbach’s alpha values were within our a priori defined boundaries and test retest reliability above the threshold; ICCs of the domain scores were between 0.69–0.87, of the subscales between 0.43–0.92.

**Table 2:** Domain-specific summary and subscale scores of the German version of the EPIC at baseline and 6 weeks follow-up and results on internal consistency and reproducibility^a^

EPIC domains	No. of items	Cronbach’s alpha reliability coefficient^a^(*n* = 92)	Test-retest reliability (ICC, 95% CI) (*n* = 44)
Domain Summary Scores			
Urinary	12	0.87	0.79 (0.65–0.88)
Bowel^b^	14	0.80	0.79 (0.64–0.88)
Sexual^b^	13	0.92	0.87 (0.77–0.93)
Hormonal	11	0.81	0.69 (0.50–0.82)
Urinary subscales			
Function	5	0.64	0.43 (0.16–0.65)
Bother	7	0.85	0.81 (0.68–0.89)
Incontinence	4	0.82	0.54 (0.30–0.72)
Irritation/obstruction	7	0.81	0.82 (0.69–0.90)
Bowel subscales			
Function^b^	7	0.51	0.78 (0.63–0.88)
Bother	7	0.81	0.77 (0.62–0.87)
Sexual subscales			
Function ^b^	9	0.92	0.92 (0.87–0.96)
Bother^b^	4	0.85	0.68 (0.47–0.81)
Hormonal subscales			
Function	5	0.58	0.61 (0.38–0.76)
Bother	6	0.69	0.65 (0.43–0.79)

*Abbreviations: EPIC* Expanded Prostate Cancer Index Composite, *ICC* intraclass correlation coefficient

^a^Cronbach’s alpha calculated using baseline scores

^b^Missing values: Bowel summary: 1, Sexual summary: 1, Bowel function: 2, Sexual function: 5, Sexual bother: 1

### Construct (convergent) validity

Tables 3 and 4 show correlation coefficients between the EPIC domain scores and the other validation instruments according to whether they fulfilled our a priori assumptions regarding strength of correlation. Cross-sectional correlations (Table 3) between the EPIC and the EORTC QLQ-PR25 domains ranged from 0.14–0.79. Correlations between the EPIC domains and the Feeling Thermometer and the SF-36 domains were weaker and ranged from 0.06–0.50 and 0.08–0.72, respectively. In most cases, correlations were stronger at 6 weeks follow-up than at baseline. Longitudinal correlations (Table 4) between the change scores of the EPIC and EORTC QLQ-PR25 domains were all > 0.5. Change score correlations between the EPIC domains and the other validation instruments were weaker and ranged from 0.07–0.50.

**Table 3:** Correlation coefficients^a^ for the EPIC domain summary scores with validation instruments at baseline and follow-up (n = 92^b^)

	Baseline				6 weeks follow-up			
	EPIC domain summary scores				EPIC domain summary scores			
	Urinary	Bowel	Sexual	Hormonal	Urinary	Bowel	Sexual	Hormonal
EORTC PR-25								
Urinary symptoms	**−0.70**				**−0.79**			
Bowel symptoms		−0.42				**−0.64**		
Hormonal treatment				**−0.58**				**−0.70**
Sexual activity			**0.68**				0.14	
Sexual functioning			**0.66**				0.25	
Feeling Thermometer^b^	0.12	0.18	0.06	**0.37**	**0.37**	0.28	0.27	**0.50**
SF-36								
Physical Functioning	**0.40**				**0.47**			
Social Functioning	0.14	0.13			**0.49**	0.19		
Bodily Pain		0.22				0.56		
Role Emotional			0.08	0.61			**0.36**	0.62
General Health				**0.47**				0.59
Vitality				0.60				0.72

*Abbreviations: EPIC* Expanded Prostate Cancer Index Composite, *EORTC PR25* European Organization for the Research and Treatment of Cancer Quality of Life Questionnaire–Prostate 25; Hormonal treatment = Hormonal treatment-related symptoms

^a^Correlation coefficients according to distribution: Spearman’s rank correlation coefficients for not-normally distributed scores at baseline and 6 weeks follow-up. Correlation coefficients are boldface font when they met our assumptions and normal font when they were higher or lower than expected

^b^Missing values: Correlations between EPIC and EORTC PR-25 domains: 3 to 13, except for EPIC sexual and EORTC PR25 sexual functioning domains (baseline = 23/follow-up = 57); Correlations between EPIC domains and Feeling Thermometer: baseline = 24–25/follow-up = 9–10; Correlations between EPIC and SF-36 domains: all time points = 0–2

**Table 4:** Correlation coefficients^a^ for the EPIC domain summary scores with validation instruments between change scores from baseline to follow-up (n = 92^b^)

	Change from baseline to follow-up			
	EPIC domain summary scores			
	Urinary (95% CI)	Bowel (95% CI)	Sexual (95% CI)	Hormonal (95% CI)
EORTC PR-25				
Urinary symptoms	**−0.69 (− 0.79, − 0.56)**			
Bowel symptoms		**−0.53 (− 0.67, − 0.35)**		
Hormonal treatment				**−0.54 (− 0.68, − 0.36)**
Sexual activity			**0.58 (0.41, 0.71)**	
Sexual functioning			**0.55 (0.24, 0.76)**	
Feeling Thermometer^b^	0.27 (0.03, 0.48)	0.07 (−0.18, 0.21)	0.21 (−0.04, 0.43)	**0.50 (0.29, 0.66)**
SF-36				
Physical Functioning	0.28 (0.08, 0.46)			
Social Functioning		0.08 (−0.13, 0.28)		
Bodily Pain		**0.34 (0.14, 0.51)**		
Role Emotional	**0.30 (0.10, 0.48)**		0.20 (−0.01, 0.39)	**0.45 (0.27, 0.60)**
General Health				**0.42 (0.23, 0.58)**
Vitality				**0.48 (0.30, 0.62)**

*Abbreviations: EPIC* Expanded Prostate Cancer Index Composite, *EORTC PR25* European Organization for the Research and Treatment of Cancer Quality of Life Questionnaire–Prostate 25; Hormonal treatment = Hormonal treatment-related symptoms. CI=Confidence Interval

^a^Correlation coefficients according to distribution: Pearson correlations coefficients for normally distributed change scores. Correlation coefficients are boldface font when they met our assumptions and normal font when they were higher or lower than expected

^b^Missing values: Correlations between EPIC and EORTC PR-25 domains: 3 to 13, except for EPIC sexual and EORTC PR25 sexual functioning domains (=62); Correlations between EPIC domains and Feeling Thermometer: change = 27–28; Correlations between EPIC and SF-36 domains: all time points = 0–2

### Responsiveness to change and MID

The SRMs of the EPIC change scores were − 1.51 for the urinary domain, − 0.62 for the bowel, − 1.91 for the sexual and − 0.26 for the hormonal domain. Table 5 shows the mean changes in the EPIC domains according to the global ratings of health state change. Table 6 summarises the anchor and distribution based estimates. We established the MID by triangulation and suggest a MID of 10 for the urinary domain, 4 for the bowel domain, 12 for the sexual domain and 6 for the hormonal domain. Additional file 1: Tables S1 and S2 show the results for the EPIC subscales; in summary, we established for the urinary subscales MID between 9 and 12, for the bowel subscales 4 and 5, for the sexual subscales 11 and 13, and for the hormonal subscales 6 and 7 (Additional file 1).

**Table 5:** Mean changes in the EPIC domains according to global rating of health state change at follow-up

Global rating of change of state of health by treatment^a^	EPIC Urinary domain	EPIC Bowel domain	EPIC Sexual domain	EPIC hormonal domain
	m (SD)	m (SD)	m (SD)	m (SD)
Worsened much (*n* = 8)	−41.6 (21.3)	−15.0 (17.8)	−56.0 (26.5)	−17.3 (11.5)
Worsened (*n* = 40)	− 33.2 (18.4)	−7.7 (9.8)	− 49.0 (19.2)	− 5.7 (14.1)
Remained the same (*n* = 31)	−21.8 (15.7)	− 5.1 (9.9)	−36.0 (24.2)	0.8 (5.5)
Improved (*n* = 6)	− 21.5 (15.2)	−4.5 (5.0)	−36.0 (23.1)	0.8 (11.1)
Improved much (n = 6)	−16.2 (20.1)	4.3 (8.1)	−41.3 (25.8)	9.8 (15.0)

*Abbreviations: EPIC* Expanded Prostate Cancer Index Composite

^a^Global rating of health state chance on the 5-point Likert scale ranging from −2 (my health state worsened much) to + 2 (my health state improved much)

**Table 6:** Anchor- and distribution-based estimates of the minimal important difference for the EPIC summary domains (*n* = 92^a^)

Anchor based approaches				
	EPIC Urinary domain	EPIC Bowel domain	EPIC Sexual domain	EPIC hormonal domain
Global rating of health state change	11.4	2.6	13	6.5
Distribution based approaches^c^				
SEM^a^	5.3	3.9	7.1	5.7
Cohen’s effect size	9.4	5.5	11.5	6.5
Empirical rule effect size	9.0	5.3	11.0	6.2
0.5 times SD	6.0	3.9	10.2	5.7

*Abbreviations: EPIC* Expanded Prostate Cancer Index Composite, *SEM* Standard error of measurement

^a^Missing values: 0–2; SEM approach based on the test-retest subsample of 44 participants

^b^Averaged difference in mean change score in EPIC domains between those who rated their health state as “worsened” and “remained the same”

^c^SEM = SD at baseline*square root[1- intraclass correlation coefficient]); Cohen’s effect size = 0.5*SD of change score; empirical rule effect size = 0.08*6*SD of change score); 0.5*SD at baseline

## Discussion

With this study we provide a culturally adapted and thoroughly validated German version of the widely used EPIC 50-item questionnaire. The German EPIC showed good internal consistency, reproducibility and construct validity and was responsive to detect changes in patients with localized prostate cancer after radical prostatectomy. We established an MID of 10 for the urinary, of 4 for the bowel, of 12 for the sexual and of 6 for the hormonal EPIC domains, respectively.

Compared to the participants of the original American development study [5] and some translation studies [7, 9], the patients from our sample achieved slightly higher but similar EPIC scores at baseline. The exception was the sexual domain and subscales, for which our patients scored much higher, indicating a better HRQL in sexual aspects before treatment compared to the other populations.

All of the domain and most of the subscale scores fulfilled the a priori defined thresholds for consistency and test-retest reliability. The few exceptions were the urinary function subscale, which reached an insufficient reproducibility, and, together with the bowel and hormonal function subscales, also a low reliability. Interestingly, the test-retest reliability and internal consistency values of the EPIC domains and subscales in our study were very similar to those presented in the original development and validation study [5].

The usually moderate to strong correlations between the EPIC and the corresponding EORTC QLQ-PR25 domains suggest good convergent construct validity and confirm that the domains of the two questionnaires reflect very similar constructs of HRQL in prostate cancer. The weaker correlations with selected and more generic SF-36 domains and the generic Feeling Thermometer were expected. Interestingly, the EPIC hormonal domain showed mostly the highest correlations with the more generic scales.

As expected, the urinary and sexual domains were much more responsive to the treatment than the bowel and hormonal domains. Radical prostatectomy predominantly affects sexual and urinary aspects of HRQL, especially soon after surgery. In contrast, other therapies such as radiotherapy would have affected rather bowel and hormonal components.

To our knowledge, we propose for the first time MID for the EPIC 50-item version. This is surprising, since there is growing awareness of the fact that outcome measurements need to be able to detect clinically relevant changes when used for evaluative purposes. MIDs have already been suggested for the two EPIC short form versions. The EPIC-26 [11] uses the same scoring system as the EPIC 50-item and retained the domain structure (only the urinary domain dropped and using the two urinary subscales urinary incontinence and urinary irritation/obstruction). Our suggested MIDs were mainly in the range of the established MIDs for the EPIC-26 (6–9 for the urinary incontinence, 5–7 for the urinary irritation/obstruction, 4–6 for bowel, 10–12 for sexual and 4–6 for the hormonal domain) [28]. The scores of the 16-item EPIC-CP [12] domains range from 0 to 12 and, therefore, the MIDs are not comparable. However, also for this 16 item version, the MID was highest estimated for the sexual domain (1.6) and lowest for the vitality/hormonal (1.0) (MID for other domains: 1.3 for urinary irritation/obstruction, 1.2 for bowel, 1.0 for urinary incontinence) [29].

Strengths of our study are the rigorous adherence to the international ISPOR guidance for the translation and validation of patient reported outcomes. The cultural adaption also took differences in mentalities between the German speaking countries into consideration and resulted in an instrument applicable in all of them. Furthermore, the assessments took place in a prospective cohort study with a priori defined hypothesis regarding results.

One limitation of the validation part of our study is that we included patients who underwent radical prostatectomy only and focused on short-term changes (6 weeks after treatment) to assess responsiveness and MID of the EPIC. As already stated and expected, the HRQL aspects of the domains urinary and sexual are more affected by this kind of treatment than the bowel and hormonal domains, which might challenge the generalizability of our results to prostate patient undergoing other treatments such as external radiation or hormonal deprivation therapy, experiencing other side effects. However, it would be interesting to replicate the analyses in prostate cancer patients undergoing other treatments, particularly the assessment of the MID in these populations. Another limitation is that we used the anchors “worsened” and “remained the same” of the global rating of health state chance variable as anchor based approach to establish the MIDs. This implies that we assume the differences between “worsened” and “remained the same” to reflect to be minimally important for patients, which we cannot be sure about. Unfortunately, the anchor EORTC QLQ-PR25 resulted in somewhat implausible values, probably due to the transformation of both instruments to 0–100 scales and the reverse scaling of some counterpart domains. An additional limitation is that for the method we used to test cross-sectional and longitudinal construct validity, correlation coefficients, the sample size of 92 patients is rather small. However, the consequence of a smaller sample size is not different correlation coefficients (which depends more on how the population is selected) but that the estimates are more imprecise, i.e. that the confidence intervals around correlation coefficients are wider compared to confidence intervals of correlation coefficients based on larger sample sizes.

## Conclusions

The German version of the EPIC showed to be a reliable, valid and responsive instrument to detect changes in HRQL components due to prostate cancer treatment. With the suggested MIDs we provide interpretation to what extent changes in HRQL are clinically relevant for patients. Hence, study results are of interest beyond German speaking countries.

## Additional files

Additional file 1 **Table S1**: Mean changes in the EPIC subscales according to global rating of health state change at follow-up. **Table S2**: Anchor- and distribution-based estimates of the minimal important difference for the EPIC subscales (*n* = 92*). (DOCX 21 kb)

Additional file 2 The Expanded Prostate Cancer Index Composite (EPIC). (PDF 45 kb)

## Abbreviations

EORTC QLQ-PR25: The European Organization for the Research and Treatment of Cancer Quality of Life Questionnaire–Prostate 25
EPIC: Expanded Prostate Cancer Index Composite
HRQL: Health related quality of life
MID: Clinically minimal important difference
PCa: Prostate Cancer
SF-36: 36-Item Short Form Health Survey
