# Supplementary material for: The German version of the Expanded Prostate Cancer Index Composite (EPIC): translation, validation and minimal important difference estimation
Source: Health Qual Life Outcomes. 2018 Feb 20;16:36. doi: 10.1186/s12955-018-0859-1 (PMC5819270; doi:10.1186/s12955-018-0859-1)
Supplement: Supplementary file 1 — Table S1: Mean changes in the EPIC subscales according to global rating of health state change at follow-up. Table S2: Anchor- and distribution-based estimates of the minimal important difference for the EPIC subscales (n = 92*). (DOCX 21 kb) [file 12955_2018_859_MOESM1_ESM.docx]

**Supplemental material**

**Table S1: Mean changes in the EPIC subscales according to global rating of health state change at follow-up**

| Global rating of change of state of health by treatment* | **Urinary** **function** subscale  m (SD) | **Urinary** **bother** subscale  m (SD) | **Urinary** **incontinence** subscale  m (SD) | **Urinary** **irritation / obstruction** subscale  m (SD) | **Bowel function** subscale  m (SD) | **Bowel** **bother** subscale  m (SD) | **Sexual** **function** subscale  m (SD) | **Sexual** **bother** subscale  m (SD) | **Hormonal** **function** subscale  m (SD) | **Hormonal** **bother** subscale  m (SD) |
| --- | --- | --- | --- | --- | --- | --- | --- | --- | --- | --- |
| Worsened much (n=8) | -54.2 (25.6) | -32.6 (20.6) | -74.8 (29.1) | -20.1 (18.3) | -10.7 (13.1) | -19.2 (24.0) | -64.3 (20.4) | -45.3 (42.9) | -21.9 (15.1) | -13.5 (9.9) |
| Worsened (n=40) | -46.4 (20.1) | -23.8 (20.4) | -63.0 (24.5) | -15.7 (19.2) | -6.7 (11.8) | -8.6 (11.2) | -52.4 (20.4) | -41.7 (26.4) | -7.5 (16.6) | -4.5 (13.3) |
| Remained the same (n=31) | -36.2 (21.9) | -11.5 (15.0) | -49.0 (25.6) | -6.8 (18.4) | -3.8 (11.4) | -6.3 (10.8) | -39.7 (23.7) | -29.6 (36.2) | 0.0 (7.5) | 1.4 (6.6) |
| Improved (n=6) | -38.3 (14.1) | -9.3 (18.3) | -59.4 (21.8) | 0.2 (17.4) | -4.8 (3.7) | -4.2 (6.9) | -37.1 (23.3) | -33.3 (26.1) | -3.3 (11.3) | 4.2 (13.2) |
| Improved much (n=6) | -23.9 (27.6) | -10.7 (16.9) | -39.2 (42.3) | -2.4 (8.4) | 0.6 (5.3) | 7.9 (11.7) | -38.0 (10.0) | -31.3 (43.8) | 10.8 (15.3) | 9.0 (16.3) |

*Abbreviations:* EPIC = Expanded Prostate Cancer Index Composite.
* Global rating of health state chance on the 5-point Likert scale ranging from -2 (my health state worsened much) to +2 (my health state improved much)

**Table S2: Anchor- and distribution-based estimates of the minimal important difference for the EPIC subscales (n=92*)**

| **Anchor based approaches** | | | | | | | | | | |
| --- | --- | --- | --- | --- | --- | --- | --- | --- | --- | --- |
|  | **Urinary function subscale**  m (SD) | **Urinary** **bother subscale**  m (SD) | **Urinary incontinence subscale**  m (SD) | **Urinary irritation / obstruction subscale**  m (SD) | **Bowel** **function subscale**  m (SD) | **Bowel** **bother subscale**  m (SD) | **Sexual** **function subscale**  m (SD) | **Sexual** **bother subscale**  m (SD) | **Hormonal function subscale**  m (SD) | **Hormonal** **bother subscale**  m (SD) |
| Global rating of health state change | 10.2 | 12.3 | 14 | 8.9 | 2.9 | 2.3 | 12.7 | 12.1 | 7.5 | 5.9 |
| **Distribution based approaches#** | | | | | | | | | | |
| SEM* | 6.4 | 6.8 | 7.2 | 5.3 | 4.2 | 4.3 | 6.9 | 14.2 | 7.6 | 5.7 |
| Cohen’s effect size | 11.0 | 9.7 | 13.7 | 9.4 | 5.7 | 6.6 | 11.2 | 16.4 | 7.7 | 6.1 |
| Empirical rule effect size | 10.6 | 9.4 | 13.1 | 9.0 | 5.5 | 6.4 | 10.8 | 15.7 | 7.4 | 5.9 |
| 0.5 times SD | 4.8 | 7.6 | 6.7 | 6.5 | 4.3 | 4.5 | 10.9 | 11.9 | 6.3 | 5.6 |
| **Proposed MID** | | | | | | | | | | |
|  | **10** | **11** | **12** | **9** | **4** | **5** | **11** | **13** | **7** | **6** |

*Abbreviations:* EPIC = Expanded Prostate Cancer Index Composite; SEM=Standard error of measurement.
* Missing values: 0-2; SEM approach based on the test-retest subsample of 44 participants

** Averaged difference in mean change score in EPIC domains between those who rated their health state as “worsened” and “remained the same”

# SEM=SD at baseline*square root[1- intraclass correlation coefficient]); Cohen’s effect size=0.5*SD of change score; empirical rule effect size=0.08*6*SD of change score); 0.5*SD at baseline
