# Supplementary material for: The German version of the Expanded Prostate Cancer Index Composite (EPIC): translation, validation and minimal important difference estimation
Source: Health Qual Life Outcomes. 2018 Feb 20;16:36. doi: 10.1186/s12955-018-0859-1 (PMC5819270; doi:10.1186/s12955-018-0859-1)
Supplement: Supplementary file 2 — The Expanded Prostate Cancer Index Composite (EPIC). (PDF 45 kb) [file 12955_2018_859_MOESM2_ESM.pdf]

EPIC

The Expanded Prostate Cancer Index Composite

Fragebogen zur Lebensqualitätserfassung bei Prostatakrebspatienten

Der vorliegende Fragebogen hat zum Ziel, die Lebensqualität von Prostatakrebspatienten zu erfassen. Damit wir eine möglichst genaue Messung erhalten, ist es wichtig, dass Sie alle Fragen ehrlich und vollständig beantworten.

Wir möchten Sie darauf hinweisen, dass die Informationen dieser Studie so wie alle medizinischen Unterlagen streng vertraulich behandelt werden.

Heutiges Datum (tragen Sie bitte das Datum ein, an dem Sie den Fragebogen ausgefüllt haben):

Tag ________ Monat ________ Jahr ________

Name (freiwillig): ____

Geburtsdatum (freiwillig): Tag __________ Monat ___________ Jahr __________

BESCHWERDEN BEIM URINIEREN

Dieser Abschnitt befasst sich mit Ihren Beschwerden beim Urinieren. Berücksichtigen Sie bitte nur die **letzten 4 Wochen**.

1. Wie oft haben Sie in den **letzten 4 Wochen** ungewollt Urin verloren?

   Mehr als einmal pro Tag 1
   Ungefähr einmal pro Tag 2
   Mehr als einmal pro Woche .... 3 (Eine Zahl einkreisen)
   Ungefähr einmal pro Woche 4
   Selten oder nie 5

2. Wie oft hatten Sie in den **letzten 4 Wochen** Blut im Urin?

   Mehr als einmal pro Tag 1
   Ungefähr einmal pro Tag 2
   Mehr als einmal pro Woche .... 3 (Eine Zahl einkreisen)
   Ungefähr einmal pro Woche 4
   Selten oder nie 5

3. Wie oft verspürten Sie in den **letzten 4 Wochen** Schmerzen oder ein Brennen beim Urinieren?

   Mehr als einmal pro Tag 1
   Ungefähr einmal pro Tag 2
   Mehr als einmal pro Woche .... 3 (Eine Zahl einkreisen)
   Ungefähr einmal pro Woche 4
   Selten oder nie 5

4. Welche der folgenden Aussagen beschreibt Ihre Kontrolle über die Blase in den **letzten 4 Wochen** am besten**?**

   Keinerlei Kontrolle über die Blase (unkontrollierter Urinverlust)... 1
   Häufiges Tröpfeln... 2 (Eine Zahl einkreisen)
   Gelegentliches Tröpfeln... 3
   Vollständige Kontrolle über die Blase................................. 4

EPIC 2.2002 Copyright 2002. The University of Michigan. Alle Rechte vorbehalten.

5. Wie viele Einlagen oder Windeln haben Sie in den **letzten 4 Wochen** normalerweise pro Tag wegen unfreiwilligem Urinverlust benötigt?

   Keine.. 0
   1 pro Tag.. 1
   2 pro Tag.. 2 (Eine Zahl einkreisen)
   3 oder mehr pro Tag 3

6. Ein wie grosses Problem hatten Sie mit den folgenden Beschwerden in den **letzten 4 Wochen**? (In jeder Zeile eine Zahl einkreisen)

	Kein Problem	Sehr kleines Problem	Kleines Problem	Mittelschweres Problem	Grosses Problem
a. Tröpfeln oder Urinverlust……………………..	0	1	2	3	4
b. Schmerzen oder Brennen beim Urinieren (Wasserlassen)…………………….	0	1	2	3	4
c. Blut im Urin……………………………………	0	1	2	3	4
d. Schwacher Harnstrahl oder unvollständige Blasenentleerung……………	0	1	2	3	4
e. Aufwachen wegen Harndrangs………………	0	1	2	3	4
f. Häufiger Harndrang tagsüber………………..	0	1	2	3	4

7. Wie würden Sie generell Ihre Beschwerden oder Probleme beim Urinieren in den **letzten 4 Wochen** beschreiben?

   Kein Problem 1
   Sehr kleines Problem 2
   Kleines Problem .... 3 (Eine Zahl einkreisen)
   Mittelschweres Problem 4
   Grosses Problem 5

EPIC 2.2002 Copyright 2002. The University of Michigan. Alle Rechte vorbehalten.

BESCHWERDEN BEIM STUHLGANG

Dieser Abschnitt befasst sich mit Ihren Beschwerden beim Stuhlgang und Bauchschmerzen. Bitte berücksichtigen Sie nur die **letzten 4 Wochen**.

8. Wie oft verspürten Sie in den **letzten 4 Wochen** starken Stuhldrang, ohne dass es tatsächlich zu Stuhlabgang kam?

Mehr als einmal pro Tag 1
Ungefähr einmal pro Tag 2
Mehr als einmal pro Woche .... 3 (Eine Zahl einkreisen)
Ungefähr einmal pro Woche 4
Selten oder nie 5

9. Wie oft hatten Sie in den **letzten 4 Wochen** unkontrollierten Stuhlabgang?

Mehr als einmal pro Tag 1
Ungefähr einmal pro Tag 2
Mehr als einmal pro Woche .... 3 (Eine Zahl einkreisen)
Ungefähr einmal pro Woche 4
Selten oder nie 5

10. Wie oft hatten Sie in den **letzten 4 Wochen** breiigen oder wässrigen Stuhlgang?

Nie.. 1
Selten 2
In ungefähr der Hälfte der Fälle .... 3 (Eine Zahl einkreisen)
Meistens.. 4
Immer 5

11. Wie oft hatten Sie in den **letzten 4 Wochen** blutigen Stuhlgang?

Nie.. 1
Selten 2
In ungefähr der Hälfte der Fälle .... 3 (Eine Zahl einkreisen)
Meistens.. 4
Immer 5

EPIC 2.2002 Copyright 2002. The University of Michigan. Alle Rechte vorbehalten.

12. Wie oft verspürten Sie in den **letzten 4 Wochen** Schmerzen beim Stuhlgang?

Nie.. 1
Selten 2
In ungefähr der Hälfte der Fälle .... 3 (Eine Zahl einkreisen)
Meistens.. 4
Immer 5

13. Wie häufig hatten Sie an einem normalen Tag in den **letzten 4 Wochen** Stuhlgang?

Zweimal oder weniger.............................. 1
Dreimal bis viermal................................... 2 (Eine Zahl einkreisen)
Fünfmal oder mehr................................... 3

14. Wie oft hatten Sie in den **letzten 4 Wochen** krampfartige Schmerzen im Bauch, Beckenbereich oder Enddarm?

Mehr als einmal pro Tag 1
Ungefähr einmal pro Tag 2
Mehr als einmal pro Woche .... 3 (Eine Zahl einkreisen)
Ungefähr einmal pro Woche 4
Selten oder nie 5

15. Ein wie grosses Problem hatten Sie mit den folgenden Beschwerden in den **letzten 4 Wochen**? (In jeder Zeile eine Zahl einkreisen)

	Kein Problem	Sehr kleines Problem	Kleines Problem	Mittelschweres Problem	Grosses Problem
a. Stuhldrang ohne Stuhlentleerung……………	0	1	2	3	4
b. Häufiger Stuhlgang……………………………	0	1	2	3	4
c. Wässriger Stuhlgang………………………….	0	1	2	3	4
d. Verminderte Kontrolle über den Stuhlgang...	0	1	2	3	4
e. Blutiger Stuhl…………………………………	0	1	2	3	4
f. Schmerzen im Bauch, Beckenbereich oder Enddarm……………………………….	0	1	2	3	4

EPIC 2.2002 Copyright 2002. The University of Michigan. Alle Rechte vorbehalten.

16. Wie würden Sie generell Ihre Beschwerden oder Probleme mit dem Stuhlgang in den **letzten 4 Wochen** beschreiben?

Kein Problem 1
Sehr kleines Problem 2
Kleines Problem .... 3 (Eine Zahl einkreisen)
Mittelschweres Problem 4
Grosses Problem 5

EPIC 2.2002 Copyright 2002. The University of Michigan. Alle Rechte vorbehalten.

SEXUELLE FUNKTIONSFÄHIGKEIT

Der folgende Abschnitt befasst sich mit Ihrer gegenwärtigen sexuellen Funktionsfähigkeit und sexuellen Zufriedenheit. Die meisten Fragen sind sehr persönlich. Sie helfen uns damit jedoch, die Probleme besser zu verstehen, mit denen Sie täglich konfrontiert werden. Wir erinnern Sie daran, dass DIE INFORMATIONEN DIESER UNTERSUCHUNG STRENG **VERTRAULICH** BEHANDELT WERDEN. Bitte antworten Sie ehrlich in Bezug auf die **letzten 4 Wochen**.

17. Wie schätzen Sie die folgenden Bereiche Ihrer sexuellen Funktionsfähigkeit in den **letzten 4 Wochen** ein? (In jeder Zeile eine Zahl einkreisen)

	Sehr gering bis gar nicht	Gering	Durch-schnittlich	Gut	Sehr gut
a. Ihr sexuelles Verlangen………………………	1	2	3	4	5
b. Ihre Erektionsfähigkeit………………………..	1	2	3	4	5
c. Ihre Orgasmusfähigkeit………………………	1	2	3	4	5

18. Wie würden Sie Ihre übliche Erektionsfähigkeit in den **letzten 4 Wochen** beschreiben?

Keine Erektionen... 1
Nicht stark genug für sexuelle Aktivitäten.......................... 2
Nur stark genug für Selbstbefriedigung und Vorspiel......... 3 (Eine Zahl einkreisen)
Stark genug für Geschlechtsverkehr 4

19. Wie würden Sie die Häufigkeit Ihrer Erektionen in den **letzten 4 Wochen** beschreiben?

Ich hatte nie eine Erektion, wenn ich eine wollte................................. 1
Ich hatte in weniger als der Hälfte der Fälle eine Erektion,
wenn ich eine wollte... 2
Ich hatte ungefähr in der Hälfte der Fälle eine Erektion,
wenn ich eine wollte... 3 (Eine Zahl einkreisen)
Ich hatte in mehr als der Hälfte der Fälle eine Erektion,
wenn ich eine wollte... 4
Ich hatte immer eine Erektion, wenn ich eine wollte........................... 5

EPIC 2.2002 Copyright 2002. The University of Michigan. Alle Rechte vorbehalten.

20. Wie oft sind Sie in den **letzten 4 Wochen** morgens oder in der Nacht mit einer Erektion aufgewacht?

Nie 1
Weniger als einmal pro Woche 2
Ungefähr einmal pro Woche .... 3 (Eine Zahl einkreisen)
Mehrmals pro Woche 4
Täglich 5

21. Wie oft waren Sie in den **letzten 4 Wochen** in irgendeiner Weise sexuell aktiv?

Gar nicht 1
Weniger als einmal pro Woche 2
Ungefähr einmal pro Woche .... 3 (Eine Zahl einkreisen)
Mehrmals pro Woche 4
Täglich 5

22. Wie oft hatten Sie in den **letzten 4 Wochen** Geschlechtsverkehr?

Gar nicht 1
Weniger als einmal pro Woche 2
Ungefähr einmal pro Woche .... 3 (Eine Zahl einkreisen)
Mehrmals pro Woche 4
Täglich 5

23. Wie würden Sie im Allgemeinen Ihre sexuelle Funktionsfähigkeit in den **letzten 4 Wochen** beschreiben?

Sehr schlecht 1
Schlecht 2
Mittelmässig .... 3 (Eine Zahl einkreisen)
Gut 4
Sehr gut 5

24. Ein wie grosses Problem hatten Sie mit den folgenden Bereichen Ihrer sexuellen Funktionsfähigkeit in den **letzten 4 Wochen**? (In jeder Zeile eine Zahl einkreisen)

	Kein Problem	Sehr kleines Problem	Kleines Problem	Mittelschweres Problem	Grosses Problem
a. Ihr sexuelles Verlangen…………………………	0	1	2	3	4
b. Ihre Erektionsfähigkeit…………………………	0	1	2	3	4
c. Ihre Orgasmusfähigkeit………………………..	0	1	2	3	4

EPIC 2.2002 Copyright 2002. The University of Michigan. Alle Rechte vorbehalten.

25. Wie würden Sie generell das Problem um Ihre sexuelle Funktionsfähigkeit oder den Verlust Ihrer sexuellen Funktionsfähigkeit in den **letzten 4 Wochen** beschreiben?

Kein Problem 1
Sehr kleines Problem 2
Kleines Problem .... 3 (Eine Zahl einkreisen)
Mittelschweres Problem 4
Grosses Problem 5

EPIC 2.2002 Copyright 2002. The University of Michigan. Alle Rechte vorbehalten.

HORMONELLE FUNKTION

Der folgende Abschnitt befasst sich mit Ihrer hormonellen Funktion. Bitte berücksichtigen Sie nur die **letzten 4 Wochen**.

26. Wie oft hatten Sie in den **letzten 4 Wochen** Hitzewallungen?

Mehr als einmal pro Tag 1
Ungefähr einmal pro Tag 2
Mehr als einmal pro Woche .... 3 (Eine Zahl einkreisen)
Ungefähr einmal pro Woche 4
Selten oder nie 5

27. Wie oft hatten Sie in den **letzten 4 Wochen** ein Spannungsgefühl oder Schmerzen im Brustbereich?

Mehr als einmal pro Tag 1
Ungefähr einmal pro Tag 2
Mehr als einmal pro Woche .... 3 (Eine Zahl einkreisen)
Ungefähr einmal pro Woche 4
Selten oder nie 5

28. Wie oft fühlten Sie sich in den **letzten 4 Wochen** niedergeschlagen oder deprimiert?

Mehr als einmal pro Tag 1
Ungefähr einmal pro Tag 2
Mehr als einmal pro Woche .... 3 (Eine Zahl einkreisen)
Ungefähr einmal pro Woche 4
Selten oder nie 5

29. Wie oft fühlten Sie sich in den **letzten 4 Wochen** kraftlos oder müde?

Mehr als einmal pro Tag 1
Ungefähr einmal pro Tag 2
Mehr als einmal pro Woche .... 3 (Eine Zahl einkreisen)
Ungefähr einmal pro Woche 4
Selten oder nie 5

EPIC 2.2002 Copyright 2002. The University of Michigan. Alle Rechte vorbehalten.

30. Wie hat sich in den **letzten 4 Wochen** Ihr Gewicht verändert?

5 kg oder mehr zugenommen 1
Weniger als 5 kg zugenommen 2
Keine Gewichtsveränderung..................... 3 (Eine Zahl einkreisen)
Weniger als 5 kg abgenommen 4
5 kg oder mehr abgenommen.................. 5

31. Ein wie grosses Problem hatten Sie mit den folgenden Beschwerden in den **letzten 4 Wochen**? (In jeder Zeile eine Zahl einkreisen)

	Kein Problem	Sehr kleines Problem	Kleines Problem	Mittelschweres Problem	Grosses Problem
a. Hitzewallungen…………………………………	0	1	2	3	4
b. Spannungsgefühl oder Schmerzen im Brustbereich / Vergrösserung der Brust……	0	1	2	3	4
c. Abnahme der Körperbehaarung……………...	0	1	2	3	4
d. Niedergeschlagenheit…………………………	0	1	2	3	4
e. Kraftlosigkeit / Müdigkeit………………………	0	1	2	3	4
f. Veränderung des Körpergewichts……………	0	1	2	3	4

Allgemeine Zufriedenheit

32. Wie zufrieden sind Sie im Allgemeinen mit Ihrer Prostatakrebsbehandlung?

Sehr unzufrieden...................................... 1
Unzufrieden... 2
Unschlüssig... 3 (Eine Zahl einkreisen)
Zufrieden 4
Sehr zufrieden.. 5

Vielen Dank für Ihre Mithilfe!

EPIC 2.2002 Copyright 2002. The University of Michigan. Alle Rechte vorbehalten.
